# 

**Published:** 1897-06

**Authors:** 


### CONTENTS.

ORIGINAL COMMUNICATIONS—

EXPERT TESTIMONY IN CRIMINAL CASES INVOLVING THE PLEA OF INSANITY. By John C. Olmsted, M.D., Atlanta, Ga. .......... 217

ENTERO-COLITIS OF INFANCY. By M. A. Clark, A.M., M.D., Macon, Ga.......... 222

MEDDLESOME INSTRUMENTATION IN URETHRAL DISEASES. By. W. L. Champion, M.D., Atlanta, Ga. .......... 230

HYPERTROPHY OF THE LINGUAL TONSIL. By W. E. Adams, M.D., Augusta, Ga.......... 237

THE ABORTIVE TREATMENT OF ACUTE SUPPURATIVE INGUINAL ADENITIS BY PRESSURE-BANDAGE. By Henry R. Slack, Ph.M., M.D., LaGrange, Ga.......... 239

CORRESPONDENCE—

THE BOARD OF CENSORS, MEDICAL ETHICS, NEWSPAPERS, ETC.......... 242

NOTES UPON A VISIT TO NEW YORK, PHILADELPHIA AND BALTIMORE.......... 246

EDITORIAL—

ETHICS AND THE NEWSPAPERS.......... 250

SELECTIONS AND ABSTRACTS. .......... 255

MEDICAL ITEMS.......... 284

BOOK REVIEWS.......... 286

MEDICAL ITEMS—CONTINUED.......... x, xii, xiv

ADVERTISERS' INDEX TO ADVERTISEMENTS..........xviii

